# Hospice Employees’ Perceptions of Their Work Environment: A Focus Group Perspective

**DOI:** 10.3390/ijerph17176147

**Published:** 2020-08-24

**Authors:** Rebecca H. Lehto, Carrie Heeter, Jeffrey Forman, Tait Shanafelt, Arif Kamal, Patrick Miller, Michael Paletta

**Affiliations:** 1 College of Nursing, Michigan State University, East Lansing, MI 48824, USA; 2 Department of Media and Information, Communication Arts & Sciences, Michigan State University, East Lansing, MI 48824; heeter@msu.edu; 3 Former Medical Director of Development, Karmanos Cancer Institute, Detroit, MI 48201, USA; jeffreydavidforman@gmail.com; 4 Department of Medicine, Stanford University, Stanford, CA 94304, USA; tshana@stanford.edu; 5 Department of Medicine, Duke Cancer Institute, Duke University, Durham, NC 27710, USA; arif.kamal@duke.edu; 6 Hospice of Michigan, 2366 Oak Valley Drive, Ann Arbor, MI 48103, USA; pmiller@hom.org (P.M.); mpaletta@hom.org (M.P.)

**Keywords:** focus groups, burnout, end-of-life care, quality of care, hospice care

## Abstract

Burnout in healthcare professionals can lead to adverse effects on physical and mental health, lower quality of care, and workforce shortages as employees leave the profession. Hospice professionals are thought to be at particularly high risk for burnout. The purpose of the study was to evaluate workplace perceptions of interdisciplinary hospice care workers who provide care to patients at end of life. Six focus groups and one semi-structured interview were conducted with mixed group of social workers, managers, nurses, hospice aides, chaplains, support staff, and a physician (*n* = 19). Findings from the groups depicted both rewards and challenges of hospice caregiving. Benefits included intrinsic satisfaction from the work, receiving positive patient and family feedback, and teamwork. Challenges reflected issues with workload, technology issues, administrative demands, travel-related problems, communication and interruptions, difficulties with taking time off from work and maintaining work-life integration, and coping with witnessing grief/loss. Hospice workers glean satisfaction from making meaningful differences in the lives of patients with terminal illness and their family members. It is an expected part of the job that certain patients and situations are particularly distressing; team support and targeted grief support is available for those times. Participants indicated that workload and administrative demands rather than dealing with death and dying were the biggest contributors to burnout. Participants reported episodic symptoms of burnout followed by deliberate steps to alleviate these symptoms. Notably, for all except one of the participants, burnout was cyclical. Symptoms would begin, they would take steps to deal with it (e.g., taking a mental health day), and they recovered. At an organizational level, a multipronged approach that includes both personal and occupational strategies is needed to support professional caregivers and help mitigate the stressors associated with hospice work.

## 1. Introduction

As both life expectancy and prevalence of chronic life-limiting conditions has soared, the demand for hospice services for patients requiring professional supportive care at the end of life has risen [1]. Since the movement’s advent, hospice services have been characterized by encompassing a holistic approach that fully attends to the patients’ physical, emotional, and spiritual needs as they face end of life [2]. Thus, hospice care is carried out by an engaged interdisciplinary team who synchronize delivery of a variety of services to ensure that patients and their family members last days are spent in dignity with freedom from suffering [3]. Modern hospice care is often fast-paced and characterized by transitioning caseloads, advanced technology and administrative demands [1,4]. There has been growing awareness and attention to the impact of front-line care on essential workers in the healthcare field who face ongoing encounters with death and dying, particularly in the context of an international pandemic. Research in this area has largely been conducted using survey approaches and/or individual interviews, and thus less inquiry has captured the shared experience of all interdisciplinary team members who work together to ensure optimal outcomes for patients and their family who are enrolled in hospice care [5,6,7,8,9]. Thus, the purpose of the current study was to simultaneously evaluate interdisciplinary team members perceptions regarding the rewarding and challenging aspects of hospice work, their experiences with professional burnout and stress management, and collective strategies to support employees across disciplines who are involved in hospice care.

Hospice work is recognized to be both worthwhile and demanding for employees committed to caring for patients facing death and supporting their family members [4,8]. The demands associated with hospice work can be highly challenging. Such demands include high patient acuity, heavy caseloads, the need to support distraught family members, and processing personal grief stemming from frequent encounters with death and dying [1]. While healthcare providers derive meaning and motivation via their commitment to better the lives of patients and families at end of life, they may encounter agency or institutional requirements that stifle autonomy, and may find ongoing workplace stressors such as documentation of activities exhausting to manage [9].

Occupational burnout has emerged as a widespread problem in health care across a spectrum of clinical care environments globally [1,10,11,12,13]. Burnout is characterized as an occupational syndrome stemming from chronic workplace stress that manifests in the form of mental and physical exhaustion, cynicism and emotional distancing from the work environment, lowered perceptions of professional competence, and increasing negativity about work [1,12]. Much of the evaluation of occupational burnout has been conducted among healthcare professionals such as nurses and physicians who work in oncology and palliative care [11,13,14]. This work has utilized surveys such as the Maslach Burnout Inventory which evaluates levels of emotional exhaustion, depersonalization and perceptions of professional accomplishment [1,14]. Untreated burnout can contribute to a host of adverse problems for the health organization including absenteeism, compromised work performance, reduced quality of care, and retention issues [3]. Further, the presence of burnout in a health care setting impacts other employees adversely as they may encounter workplace negativity and turnover of colleagues when attrition occurs [10].

Hospice services include a broad spectrum of care such as symptom management, supportive patient care, financial and instrumental support, spiritual care, and grief support [2]. Therefore optimal end of life care relies upon seamless coordination of an interdisciplinary team to ensure patients face death with dignity, support, and comfort [3]. However, limited research has examined perceptions of the hospice work experience from an interdisciplinary perspective. Studies have found that physicians and nurses who work in palliative care and hospice can experience substantive professional burnout [3]. However, there is less research that has engaged the lived perceptions of health care assistants who provide ongoing direct patient care to the dying. Further, the experiences and perspectives of chaplains who provide spiritual care and of hospice social workers that coordinate complex caseloads have not been well documented. While group-based social research, such as the incorporation of focus groups has grown increasingly popular to gain deeper perspectives around a topic of interest, much of this research has been conducted homogeneously among similar groups, detracting from the ability to glean insights into exchanges between disciplines [15].

Organizations such as hospice have made strides in program evaluation recognizing the important roles of various stakeholders to ascertain needs and to better deliver services to their care recipients [2]. Focus groups are a strong qualitative methodology to explore diverse challenges, gain deep insight into experiences, and foster program evaluation [16,17] including in healthcare environments [15,18]. The focus group format provides an opening for participants with shared background to dialogue about their experiences while providing important information that can be used to enhance the workplace environment [19]. An important strength of the focus group methodology is that it also offers the essential space for professional hierarchies and multi-disciplinary divisions to be surmounted as professionals across roles discuss perceived problems and opportunities [15]. A minimum of three to four focus groups are recommended to ensure adequate coverage of the desired content with the aim of attaining content saturation with no new patterns and information provided [20]. The purpose of the present study was to evaluate hospice employees’ perceptions including rewarding and challenging aspects of their work, perspectives on burnout, personal approaches to stress management and insights regarding organizational strategies to reduce occupational distress. The five targeted research questions were: What are the perceived positive aspects associated with hospice employment? What are the perceived challenges associated with hospice employment? How do employees perceive burnout? What strategies do hospice workers use to combat burnout and manage stress? What program recommendations do hospice workers have for organizations to reduce burnout?

## 2. Materials and Methods

**Figure 1:**
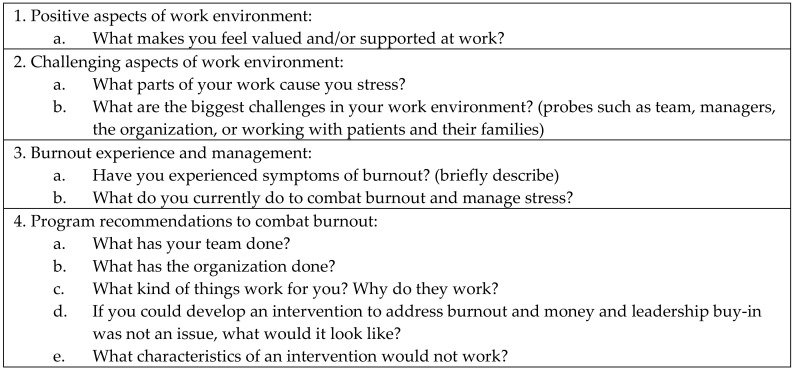
Brief Summary of Focus Group Interview Questions.

The associated University Institutional Review Board approved all research procedures prior to the study execution [STUDY00003263]. The researchers contacted employees from a large Midwestern hospice organization via email to query their interest relative to participating in the focus group study. Of 96 employees who volunteered, 23 were selected for participation balancing length of time employed at the hospice organization, role, and geographic area to ensure a representative sample of participants with an aim of four participants per group. Mini focus groups (less than five participants) are used when group participation may be challenging to obtain, but is necessary for the type of discussion and engagement needed [20]. Those who agreed to participate completed an on-line consent form. Pre-session planning and content for the focus groups was developed consistent with study aims and was approved by a panel of researchers, practitioners and staff from the hospice research team [17,20]. All focus groups sessions were held via video conferencing over a 90-min period across a 4-week period (October 2019–November 2019) and were audio recorded, a method that our team had already tested in previous research [21]. Established focus group rules were discussed including confidentiality, use of first names, and equal opportunity to communicate viewpoints. Focus groups questions were standardized across groups and elicited content about positive and challenging aspects of the work environment, perceptions of burnout, strategies to manage work stressors, and recommendations for organizational management (see Figure 1). Participants were compensated with a $40.00 Amazon gift card in appreciation of their service and time.

Three researchers with expertise in focus group methodology including both a nurse and a physician scientist guided the sessions. To ensure accuracy, the researchers debriefed with the volunteers and clarified any ambiguous interpretations and needs for further information. Further, the confirmability of study findings was established at the conclusion of each focus group among the researchers with discussion between the moderator and the researchers taking additional field notes. In addition, the three researchers who conducted the focus groups all independently evaluated the finalized data transcripts.

Audio recordings were transcribed verbatim. Prior to analysis, to preserve anonymity and to facilitate analysis by role, and names were replaced by role (e.g., Nurse was indicated by “N”; Social Worker by “SW”) and unique numeric identifiers were assigned to each individual, thus, the first nurse who participated was N 1, the second nurse was N 2, etc. Krueger and Casey’s [16] strategy of constant comparative analysis then guided how data were evaluated. The constant comparative analysis method provided an opportunity for the researchers to compare and contrast the data across all focus groups to determine relationship patterns in the data [16]. After transcription, a consolidated analyses of the study findings occurred. Results were systematically coded and organized into content categories as described below.

## 3. Results

The six focus groups (1st group = four participants; 2nd group = two participants; 3rd group = four participants; 4th group = three participants; 5th group = two participants; 6th group = three participants) and one semi-structured interview totaled 19 participants including five nurses, three managers, three social workers, three support staff, two chaplains, two patient care aides, and one physician). Participants were primarily women (*n* = 16, 84%). The distribution of roles and gender reflect distribution across the organization. 

Although planning including reminder emails was carefully conducted in advance of the scheduled groups, hospice work is dynamic and unforeseen circumstances can create last minute alterations in professionals’ schedules. Thus, while the semi-structured interview with one participant was unanticipated (four participants out of the invited 23 did not participate due to such unanticipated schedule conflicts), it provided triangulation as an alternative data collection strategy providing further evidence for credibility for study findings [22]. 

Participants were selected to be diverse with respect to the size of the communities they served with 11 members from large urban areas and eight from small towns or rural regions. Experience at the hospice organization was also a selection factor to ensure representation of new, medium length and long-term hospice workers. Seven focus group participants had been employed in their current position for three or less years, eight participants from four to ten years, and four participants having more than 10 years in their current positions in the hospice organization. In the following paragraphs, focus group findings are organized around the five aforementioned research questions with description of the major findings. Table 1 presents the major content categories that were derived in the study.

### 3.1. Rewarding Aspects Associated with Hospice Employment

Recurrent descriptions of the sources of joy related to hospice work centered on feeling valued and supported in their professional role by patients and their family members. Participants appreciated receiving positive feedback from patients and families. For example, a nurse (N1) stated:

“*We feel valued… My families are beyond what keeps me going all the time, because they couldn’t be more appreciative and you just don’t get that in the hospital anymore, people that really appreciate you. I love that.*”

Participants described gaining intrinsic satisfaction from having meaningful impacts on the lives of patients and their families during a profound time. Another nurse (N3) stated:

“*It is just feeling privileged enough to be in the end of these people’s lives and making sure that it’s not about dying, but it’s about living before we die. So when I can let family members own that they were there and advocating for their loved ones and I can facilitate that…*”

Further, participants from all of seven clinical roles highlighted expressions of support and appreciation from fellow employees including management, and enjoyment of being part of a cohesive team. As examples, one of the chaplains (C1) and the physician (P) articulated in respective order:

“*We have staff… awards Each month, there is a drawing—One of the nominees is picked randomly for an award. Sometimes the award is a coupon to a place or a restaurant of coffee shop. Recently the award has been CTD (coded time off). So you get half a day off. That’s really nice.*”

“*When I say that the other person on my team is going to do X, Y and Z, I know that they’re going to do it. And then to share that experience as a team and caring for these patients brings a lot of joy.*”

Participants identified the rewarding aspects derived from regular feedback and interaction. A manager (M1) stated:

“*You got to tell the nurse that you appreciate them coming in two hours early, or staying over two hours later, or working on the weekend when they were supposed to be off… say thank you, send an email and say you appreciate your staff…*”

### 3.2. Perceived Challenges Associated with Hospice Employment

Focus group participants expressed a myriad of challenges relative to their hospice work. These challenges included the ongoing burden of heavy caseloads, additional workload stressors such as unpredictable assignments and schedules, communication issues, work interruptions, travel-related issues and weather problems (during winter season), difficult family issues, administrative demands, problems with technology, difficulties with being able to go on vacation or taking time off, competing personal life demands, taking ‘work’ home, managing loss and grief associated with the death of patients, and role ambiguity. Examples from the focus groups follow in this regard.

#### 3.2.1. Heavy Caseloads

Caseloads are widely different by role due to the nature of the work, with social workers and chaplains carrying larger numbers of patients than nurses and aides. Despite these differences, many of the challenges reported were remarkably similar. Workload is intense; often there is not time for lunch (unless while driving) or even to use the bathroom. For example, one of the Aides (A1) described:

“*I’m covering two areas and I feel like I’m not giving each area the care… I’m giving my 100%, but I feel like I’m rushing from one area to the other…I am covering an area that’s an hour away from where I’m at, and so I have to work my schedule in … and I just feel like I can’t focus on my area. I feel like I’m rushing through each area and I don’t feel like I did my best even though I give my best.*”

#### 3.2.2. Additional Workload Stressors

Different roles reported different challenges. Many of the hospice worker roles are salaried, officially paid and expect to work 40 h per week, but often ending up putting in additional hours without additional compensation. Managers are also salaried but have less expectation of “only” working 40 h. On the other hand, aides are paid overtime when they work more than 8 h, but are discouraged from doing so. These differences led to different pressures.

Given a dynamic work environment where hospice care is provided at the patient home, 24/7 hospice consultation is provided, new patients can be admitted to hospice at any time, and the unpredictable course of dying, participants reported that a day of hospice work almost never unfolds the way it appeared like it would when the day began. Participants described the impact of the need to accommodate additional workload when fellow co-workers are ill or take time off, when a new admission is added to their day, when planned visits require more time than scheduled, and patient and family-related issues. Hospice workers who try to efficiently manage their schedules and get work done quickly often pay the unexpected price of being asked to take on extra work that day. Participants discussed stressors such as unpredictable assignments and schedules. It is not unusual for new admissions and/or patient emergencies to occur toward the end of a shift, interrupting the professionals’ personal plans if at the last minute they learn they have to work late. Such schedule interruptions do not occur every day, but when they do occur they can be non-negotiable. That professional is the only one there to take care of a patient in need. Agreeing to help out when emergencies arise (which happens regularly) rather than saying “no” can lead to becoming the “go-to” person for extra work. The desire to help and the need to set boundaries are challenging to reconcile, and the tension between these two urges is omnipresent. As examples, a social worker (SW1) and two nurses (N2; N4) stated:

“*There’s never any solutions presented as to what to do… It ends up being that patients are shuffled around teams, and there’s times where social workers have patients on three or four different teams, which is really hectic and very confusing.*”

“*One of my bigger stressors is that I hired in for a particular team, which has a very large area that we cover and I am sent to other teams more frequently than I’m in my own area.*”

“*I start my week out with a planner and it’s very specific as to what I’m going to do and it never goes as planned… I plan out my routine stuff and I leave gaps throughout the week so that I can fit… unscheduled visits in. But if I have a patient that has fallen, and I’ve got a really busy day… and another patient that’s throwing up and now I have a visit for a fall that I have to try to squeeze in it can get pretty hectic.*”

#### 3.2.3. Communication Issues

Communication problems may also contribute to the chaotic nature of many workdays. Interdisciplinary teams, by definition, means different roles, responsibilities, and vocabularies, all caring for the same patients, usually in separate visits or calls. Such complexity can result in unanticipated communication problems as indicated by the following nurse’s example:

“*Sometimes we’ll ask two or three different people to go see the same person. And then it’s a lot of confusion where you end up calling each other to figure out who’s actually going to see the patient. Otherwise, you’re driving, our area is quite large, so you might end up driving that extra 40 miles out of your way because of confusion and lack of communication…mostly on the manager’s part.*”

#### 3.2.4. Work Interruptions

Potential interruptions in the form of emails, text messages, and phone calls are also ongoing features of the work environment. The physician depicted:

“*Part of it is the interruptions. I try and sit down to do a task, and whether it’s meet with a patient, or family, or try and develop a policy, or even to write my notes from the patients I’ve already seen, and it just seems like my phone is constantly ringing. I’m getting pinged through emails. Some of the phone calls and whatnot, I don’t know if they’re emergent or if it’s FYI* [for your information], *until I have to stop what I’m doing, answer the phone, that could take 30 s to 10, 15 min. And then I’ve got to try and re-engage, and I feel like I waste a lot of time spinning my wheels, and I realize that’s part of the work that we do, is that with patients they’re going to call when they need something, but that has been a very frustrating aspect the last couple of months.*”

#### 3.2.5. Inclement Weather and Travel-Related Issues

Focus group participants who needed to travel wide distances to patients’ homes including those serving in remote areas had the additional challenge of driving demands and winter-related weather issues. One nurse described having a 200-mile radius to cover in a day’s work. Another nurse from a rural region reported having to get up hours before dawn to shovel snow, defrost her vehicle, and start the one-hour drive to visit a new patient, as rural road conditions were often bad. The following statement is from one of the managers [M3]:

“*We often cover multiple counties. I think another stressor to that is if a person lives an hour, hour and fifteen hour and a half away, we have that time to drive there, sometimes further for an hour, hour and a half, and then the drive time, that could be like half a day to only get in one visit. There’s a lot of balancing that we do with that.*”

#### 3.2.6. Difficult Hospice Family Issues

At times, problems can arise with family members who may not accept that their loved one is dying or who are particularly needy. Family problems also included personal safety concerns such as with large dogs, angry family members, and their episodic non-willingness to adhere to patient education needs. For example, an Aide (A2) described:

“*… I feel like when we are expected to do a lot more than what people understand, a lot of our management they don’t work in the field, so they don’t know how our visits go and they’re like, “Why were you there for an hour and a half?” Well, if you were there, you would have known why…*”

#### 3.2.7. Administrative Demands

Documentation of every visit in the EMR system needs to be completed before the end of each day. Participants described demands such as regulatory issues and compliance requirements as contributing to less time to spend with patients. As an example, one of the chaplains (C2) indicated:

“*All the documentation basically… like they started with the nursing assessments and then they sort of adapted them to the other disciplines, but they don’t adapt. So my assessments are downright silly in some parts. Every time I do a routine assessment, I’m supposed to put in the patient’s belief system. If they were Catholic last month, chances are good they’re Catholic this month…*”

#### 3.2.8. Technology Problems

Issues with technology were identified as a major concern across all the focus groups. Hospice employees who serve patients and families in more remote and rural areas experience the disruption of “no service internet zones”. The tablets that are required to be used for documentation during patient visits rely on internet connectivity. Data can be lost and need to be re-entered. An aide from a rural area depicted how after leaving the patient’s home, she would drive until cellular service reception indicator appeared and then stop her vehicle and document her visit activities before driving the rest of the way to her next patient’s home. A nurse from a small town indicated that she always carries two phones in an effort to ensure that she had some kind of internet coverage for communication and documentation. While the virtual electronic medical record (EMR) is assumed to be available in real time for nurses, aides, social workers, and other hospice professionals, connectivity issues occur, particularly in more remote areas for staff members making home visits. Resulting problems include lack of access to documentation while at the patient home and challenges with getting essential tasks done in a timely manner such as ordering medications. Unanticipated network failures can result in a loss of documentation and need to re-enter all of the information. Examples follow from two nurses (N4, N5):

“*We have no service zones. Sometimes, I turn mine into airplane mode, ensures that way and then when I have signal again, it will all load… I actually charted a four-hour visit two weeks ago. It was one of the most lengthy visits I’ve ever done, and I had so much detail in it. I went to submit it and the network went down, and it told me I lost all of it. I don’t know what happened, but I shut it and when I came back to it an hour later, everything was there again. We had a week straight where it was up and down, up and down regularly.*”

“*Your tablet is spinning around because you can’t get… get to a hotspot, do this. Everything sounds good when you’re working in an office. People mean well. If they’ve never done the job, or have never been out on the road, they don’t know what it’s like… Something might take you down the wrong road, as far as technology. You might get somewhere where you got a dead spot you can’t get phone coverage up here, especially up here. I’m in a largely rural area.*”

Another frustration associated with technology was related to wasted time. The tablets used by the staff require frequent updates. Even when updates are automatic, use of the tablet needs to wait for the update to complete. Participants described problems such as needing to call information technology (IT) and having to wait on the phone for service. Transition to use of tablets occurred at the same time as a new EMR system was adopted. Clinicians with less computer literacy and those who had been with the organization for longer than ten years seemed less happy with the new system, given they started their employment with paper-based charts, then were trained on computer-based charting, and now are using another new technology (tablets) and a new EMR system that continues to change. A support staff (SS 2) indicated:

“*There are days where we’re just constantly on the phone with IT and you’re trying to be quick and efficient and things are just getting in the way; your* [network] *can go down, which is where the charting takes place, or everything can just freeze for quite a few minutes. You can come back from lunch and one of your* [networks] *is just down, it’s gone and you have to pull it back up. So it’s very time consuming.*”

Some policy and technology challenges are interrelated. All patient visits by a hospice professional (including nurses, aides, social workers, etc.) must be documented in the EMR using the tablet system. Many participants expressed frustration that required documentation during patient visits takes away their ability to focus on the patient and family and uses up time that would otherwise be spent interacting with patient and family. For example, one of the nurses (N5) stated:

“*Everything always looks good on paper. But when you get right out there and do it, not so much sometimes… When you’re charting, you’re looking at a computer, you’re not looking at these people…These people are the sickest of the sick… We owe them our undivided attention when we’re in their house. I do my charting in the car.*”

#### 3.2.9. Taking Personal Vacation or Time off

In particular, social workers and chaplains described having increased workloads before and after vacation periods due to not having case coverage during off periods. For example, a social worker (SW1) identified:

“*We have vacation days and management encourages us to take them. But in order … for me to take…a week vacation in a month, that means I have to go see my entire caseload, which is hovering at 50-plus in three weeks rather than four weeks because there isn’t anybody that is going to be picking up visits for me while I’m gone… to have any time off, I have to work extra hard, proceeding it, and then when I come back, and that makes for very anxious vacations.*”

#### 3.2.10. Competing Personal Life Demands

Participants discussed having their personal plans stymied, such as going to a child’s sports event or a concert secondary to make work-related adjustments. Not knowing whether personal plans might need to be canceled until the end of the day, is a source of stress even when such disruptions do not occur. Wondering if it will occur and not being in control is a source of stress. These non-negotiable interruptions occurred often enough that the nurses and aides (following quotes in respective order from N3 and A1) expressed anxiety about the possibilities when personal evening plans were made.

“*What causes me a lot of stress is not knowing am I going to be mandated on the weekend. I don’t mind working late, occasionally. But…you want to be able to go to yoga class at 5:30, or you want to be able to go to your niece’s volleyball game.*”

“*When I get home, it’s like I have enough time to start dinner, make sure my kid is alive, and whatever chores I can get done, I cram it all in. As far as doctor’s appointments, it’s very hard to get into the doctor when you work Monday through Friday. It’s just hard to arrange things to make it so that you can do your wellness… You can call your boss and say, “Listen, I have a doctor’s appointment,” and now I’m taking my lunch break for my doctor’s appointment… I feel like I’ve been putting my health off for my job … I’m done at five o’clock… I get a six o’clock doctor’s appointment, I’m not guaranteed to actually be done by five o’clock because I never know from one patient to the next.*”

#### 3.2.11. Taking ‘Work’ Home

Nurses, in particular were more likely to describe challenges associated with thinking about work and checking on the status of patients during off time. When a patient gets very near death, it becomes more difficult for the clinicians caring for that patient to disconnect from work at the end of a workday. The hospice system provides 24-7 support and includes rapid response teams who cover nights and weekends. Even though other hospice workers have taken over providing care to that patient, the feeling of caring doesn’t stop. For example, a nurse (N2) described constantly thinking about her patients and checking her phone for patient updates on the weekend.

“*I think sometimes that we get vested in our families as well, and it’s hard to not look at your phone for updates. If you know somebody’s dying and it’s imminent, it’s really hard to turn that off. I’ve caught myself on vacation checking my phone to make sure certain people hadn’t passed away, or that my patients are being taken care of when I am off. I try to address all my patients’ needs before I do have days off so that they don’t have to call the call center.*”

#### 3.2.12. Managing Loss and Grief

Hospice workers’ role is to support the dying process including the grief of patients and families. Some participants described feeling challenged with finding the opportunity to grieve the loss of patients themselves. For example, one nurse (N 3) stated that she couldn’t cry when attending a ‘death’ visit. Given her role as a coordinator, she felt she needed to demonstrate emotional fortitude around family members.

“*You can’t cry during a death visit. There’s times that I have with the family, but you’re the strong one, you’re the one that has to coordinate how this is going, and you have to put your feelings aside. I feel like sometimes this job doesn’t allow you to take the time to take a pause and breathe because … or you’re getting a new patient before the body is even being taken by the funeral home.*”

#### 3.2.13. Role Ambiguity

Social workers said they sometimes felt a need to act as case manager (which is not their role) if the role was not being fulfilled. Support staff coordinators expressed that volunteers sometimes are asked to take on nurse responsibilities so roles became blurred. An example statement from a social worker (SW 1) follows:

SW 1: “*Again, this isn’t across the board, but just definitely with certain team members… where I almost have to act like the case manager in a lot of ways when certain nurses aren’t doing what they’re supposed to be doing and I have to do essentially nursing duties when I’m not a nurse.*”

### 3.3. Burnout Experiences

All the hospice clinicians described feeling episodic emotional strain. Participants stated that they recognized they were approaching burnout when they start dreading going to work. The demands from unpredictable schedules, case overload, particularly challenging patient and/or family situations, and mental and physical exhaustion can begin to compromise their ability to provide compassionate care. One of the chaplains (C2) stated:

“*As one gets more exhausted, one gets less open to being enriched by one’s patients and family members, and it becomes … a vicious cycle. You get exhausted, so you’re less open to being recharged and then you get more exhausted.*”

The hospice professionals in our study all described being able to identify when they were beginning to experience burnout symptoms and needed to practice self-care by taking breaks from their work. In other words, burnout started to show up. They took steps to address it. And those feelings eased. Burnout was episodic and recurring, not constant. A social worker (SW2) identified burnout as:

“*that feeling of dread …of going to work in the morning. It’s feeling like irrational anger with my caseload. That’s when I know things are careening off course, is when I don’t wake up and feel good about going to work.*”

Although participants associated organizational issues and not patient issues with the experiences of burnout, working with death and dying nonetheless took an emotional toll. This was expected, part of the job. Some of that emotional toll was cyclical. Certain patients affected hospice workers more deeply than others, such as due to poignancy of the circumstance or extent of suffering were more emotionally devastating and when those arose, the professional would often seek support themselves. For some employees, particularly newer employees, the emotional toll was more continuous. They reported it was generally difficult to ‘turn off’ thoughts about their patients when not at work or when on vacation. For example, one of the nurses (N3) stated:

“*…brain fatigue. At the end of a busy day or a busy stretch of working, you just feel defeated a little bit. I always have a sense of I’ve done good, but I get to the point at the end of the week, seriously, I do not want to make one more decision. I don’t care…Sleepless nights, racing thoughts, physical fatigue, emotional fatigue. That’s what burnout is.*”

### 3.4. Personal Strategies Used to Combat Burnout and Manage Stress

Hospice professionals who worked part time and/or who had more autonomy in scheduling were able to use that flexibility to help avoid burnout or to deal with burnout when it started to show up. Participants used self-care strategies such as taking personal time, yoga, meditation, exercise, spiritual practices, leaving work at work, and distraction to bolster recovery. Examples from a nurse and physician follow:

“*If I start to feel like I’m getting to that edge, I know that I need to regroup, take a weekend away, do some yoga, do some guided meditation, go for a run, play with my family members, do something away from nursing. So I think through the years I’ve learned how to hone in on that.*”

“*I very directly engage in my faith. That to me has been really helpful, especially in the midst of all the suffering that we see… so to have a good lens to process that and a feeling of being supported and…feeling called to do this work, and so being very directly engaged with my faith has been helpful.*”

One aide identified setting boundaries and being assertive about caring for herself.

“*I set boundaries for sure, 100%. Today, can you do this? No, my shift ends at 3:30. It’s going to be my last patient and I cannot help you. I just set boundaries. I just have to, because if not they will just keep adding and adding and adding and keep asking and asking and asking. This is all I can do for today. That’s it. And then I go home, and I don’t think about work. I put my tablet away. I put my phone away, and I don’t look at it until the next day.*”

Participants who had worked in hospice for longer duration and self-monitored were able to mindfully identify when things were reaching capacity appeared better able to take steps to prevent burnout symptoms from escalating. These more senior clinicians (meaning those who have worked in their current role for more than 10 years) reported they had developed practices of self-care that they learned are necessary to protect personal time, such as resisting the urge to check email on nights and weekends. As an example, a social worker identified:

“*I’ve been doing this a long time and I have learned through the years to recognize when I’m getting to the point where I need to take care of myself and I’ll work at doing that as much as I can. So I have learned to prevent that* [burnout] *from happening.*”

#### 3.4.1. Organizational Strategies Used to Combat Burnout and Manage Stress

There were a variety of hospice organization institutional strategies in place to support employees’ capacity to manage work-related stressors, such as an 800 number that employees can call to speak with grief counseling services. Different teams were reported to have different practices. These approaches included such things as monthly staff meetings that include grief support and going through the list of patients who had died and taking time to reflect on those people; employee awards, where team members nominate a fellow employee for something they’ve done well; providing staff appreciation lunches and/or food for employees, and holiday activities. Some of the disciplines held discipline-specific gatherings, either in person or via technology. Staff have recently been encouraged to take ‘windshield’ time where they sit in their car and take some quiet breaths before going to the next patient’s home.

Professional development in the form of local seminars and larger professional events were valued, especially when the organization provided financial support and attendance occurs on professional time. Some of the participants described the importance of connecting with other people in the organization who were in their discipline. For example, the chaplains valued engaging in a bi-monthly video conferences with other chaplains (see comment below). Statewide meetings, whether in person or via technology, were seen as valuable and supportive.

“*Conferences, in general, are bringing in relevant speakers. I have been benefited since I’ve been here from the Jewish Hospice and Chaplaincy Network’s annual caregiver conference…and it’s huge pick me up. You hear new ideas.*”

#### 3.4.2. Recommendations for the Organization to Reduce Burnout

Participants were wide-ranging in their recommendations for organizational involvement in employee self-care. What is appreciated by one hospice professional may not be desirable for someone else. For example, some hospice workers appreciated social events, while others found them hard to fit in. Some hospice workers enjoyed staff appreciation lunches and free food, but others were more resistant to food as an option to express appreciation. Most participants did not want burnout reduction strategies that would require time commitment outside of the hours they already work. Ideas such as being given personal time that is in addition to sick leave or vacation benefits were offered. One participant suggested offering training in yoga or guided meditation as an option (during working hours). Another idea was to offer incentives for employees to engage in self-care, such as gym memberships. For example, a social worker (SW3) identified:

“*So having some kind of self-care that’s built in, like this is what we do, this is like everybody’s doing it, and anytime you do a self-care activity you are keeping track of it and there’s incentives. So you get so many self-care points and you actually can get something… It would be fun, and it’s always good to have fun anytime you can have fun because we know how important laughter is in self-care.*”

## 4. Discussion

The focus groups provided useful information about the lived experience of hospice workers in a forum that provided inter-professional dialogue about rewards and challenges of their work environment. The rich discussion provides a foundation from which organizational strategies to enhance employee wellness can be developed.

While hospice employees identified many fulfilling positive benefits derived from their work, they also identified stressors that are potentially modifiable. Consideration of ways to combat or prevent burnout for hospice professionals needs to be grounded in understanding the sources of stress and challenges of the work [5]. The fact that patients are dying, and/or that patients and families are suffering were never mentioned as a direct source of burnout. Hospice philosophy is grounded in relieving suffering at end of life and employees who commit to care of the dying may have psychologically accepted or integrated the reality of death. For example, a qualitative study conducted with hospice workers found that death encounters were perceived as enhancing personal growth and finding personal meaning with life [6].

While there were sometimes difficult situations or people, the hospice professionals in our focus groups thrived on taking care of families and patients and reported this as the most meaningful aspect of their work. The work is emotionally demanding but meaningful. Instead, it is work overload and structural and administrative characteristics of the work that more often bring about burnout. Frequent changes in scheduling and fluctuating caseloads mean that available time for each patient is constrained and that personal life plans can be disrupted without warning. Many hospice professionals also reported feeling stress and lack of control when new or revised work protocols were implemented. Communication problems would also contribute to stress. Problematic aspects associated with these work-related stressors have been documented in earlier research among hospice stakeholders and in hospice workers internationally [2,5].

Administrative policies such as documentation and mandated training modules are a necessary component for accreditation and for ensuring that employees are compliant with organizational directives. Documentation during patient visits, though necessary, took focus away from patients and families and used up limited visit time. Employees expressed displeasure when additional job training was required to happen outside of work time. Perhaps more importantly, technical issues related to connectivity and usability made the process more burdensome than necessary. Such technology issues were identified as a problem in every focus group.

In the focus groups, burnout symptoms were episodic. Most burnout studies have been done using cross-sectional surveys with longitudinal studies suggesting fluctuations in the experience [3,4,8]. Only one of the 19 participants reported being thoroughly burned out (after decades of hospice work) and preparing to leave the profession. For the rest, burnout symptoms occurred episodically, participants instituted time off and self-care strategies and experienced recovery. For example, when the employees felt the symptoms of burnout coming on, they reported preventive or remedial actions such as personal time or restorative activities that help them emerge from the burnout experience. They then return to feeling engaged with the work of providing hospice care. Such ongoing awareness of the need to monitor the self and provide self-care has been shown to improve professional quality of life in other research [23].

A major contributor to burnout and on the other hand an essential skill for combatting burnout is the importance of setting personal boundaries such as not checking email or one’s work phone during personal time. The life and death nature of this work can easily compel thinking about work all the time. While it can be very challenging to not check on patients who are nearing death, not being able to disconnect from work negatively impacts personal time. Saying no when asked to cover the latest unexpected need feels wrong, yet setting boundaries, including saying “no”, is one of the keys to self-care.

The work environment including positive work-related conditions and perceived occupational support are associated with better perceived and objective health [24]. Instilling burnout prevention practices as part of the organizational infrastructure may yield positive benefits for employee wellness [25,26,27,28]. The organizational approaches people felt would be most helpful were diverse. More extroverted employees and those with fewer time demands from their own family situation may desire socially oriented events to reduce perceived stress. Some of the hospice teams already practice such activities with strong engagement from employees whereas other teams do not have such practices. Such differences in an organization suggest that individual managers may be in a strong position to collaboratively inquire with their team [13,28,29,30,31] and then tailor plans in accordance with what makes the best sense in this regard. Interventions that are pushed out from leadership need to be informed by evidence in the literature on what works to support specific teams [32,33,34]. While variance in individualized preferences and professional roles lead to unique challenges in developing or deciding on interventions, commonalities appeared to outweigh the differences based on the focus group findings. Having prescribed time off for self-care and mental health appeared to be a universal recommendation across groups.

Other research has identified the value of workplace interpersonal relationships for managing occupational stress [35]. The focus groups also afforded an opportunity for co-workers across disciplines and geographic regions to discuss concerns and challenges in an open format that provided a venue for employees to feel supported and listened to by management [33]. Participants expressed gratitude to their organization for supporting the focus groups. Further, they appreciated a chance to learn that others were experiencing similar challenges thus providing connection and solidarity perceptions. For example, one of the social workers expressed “It’s nice to hear from such a cross section of people and finally being validated in that you’re not crazy, or whiny… It’s nice to hear that everybody has very similar concerns that point to systemic changes needing to be made.”

### Limitations

There are inherent limitations associated with focus group research. The opinions and perspectives expressed were those of a self-selected group of employee representatives. Selection was based entirely on balancing structural factors (role, population density, and years in current position) to insure diverse perspectives were represented. The researcher who made the selections knew nothing about the volunteers other than structural factors. It is always possible that the ‘group’ itself could impact the content discussed. This issue was more than likely averted given the number of focus groups conducted. Another limitation relates to having participants initially agree to participate, but then not being present at the time of the focus groups (*n* = 4). This potentially could have resulted in a loss of valuable information and opportunity for alternative viewpoints. Further, the sample sizes in the mini-focus groups were smaller as compared to what are traditionally reported [20]. While the study provides important content, it may be context specific to one large hospice organization in a Midwest state that provides a mix of in-patient and patient home hospice care across a wide geography but may not reflect the experiences of hospice workers in all organizations. Focus group research is not generalizable and may not reflect the experiences of hospice workers in other organizations.

## 5. Conclusions

The growing demand for hospice care services has led to development of multidisciplinary teams to ensure high quality care at end of life. To avoid hospice worker burnout, it is vitally important that the perspectives, needs and challenges of hospice employees serving varying clinical care roles receive the attention of health care system management. The qualitative research described here provides insight into the challenges and rewards of hospice care as well as organizational characteristics that contribute to and mitigate burnout. Organizational efforts to mitigate excessive work demands, provide appropriate cross-coverage, facilitate self-care, and provide resources to address structural issues can improve quality of life for the providers and quality of care for patients and families.

## Figures and Tables

**Table 1:** Content categories and subcategories.

Categories	Subcategories
Rewarding Aspects	Feeling valued by patients and families
	Intrinsic satisfaction for the type of work
	Management recognition and support
	Teamwork
	Regular feedback and interaction
Perceived challenges	Heavy caseloads
	Additional work stressors (including unanticipated, and/or unpredictable assignments and schedules)
	Communication Issues
	Work interruptions
	Inclement weather and travel-related issues
	Difficult hospice family issues
	Administrative demands
	Technology problems Taking personal vacation or time off
	Competing personal demands
	Taking ‘work’ home
	Managing loss and grief
	Role ambiguity
Strategies to combat burnout and manage stress [personal (p) and organizational (o)]	Boundary-setting (p)
	Self-care (p)
	Mindfulness (p)
	Grief support (o) Staff appreciation (o) Continuing education (o)
